# Arthroscopic Screw Fixation Technique for an Isolated Avulsion Fracture of the Lesser Tuberosity

**DOI:** 10.1016/j.eats.2025.103711

**Published:** 2025-06-30

**Authors:** Enver İpek, Yusuf Sülek

**Affiliations:** Department of Orthopedics and Traumatology, University of Health Sciences Türkiye, Şişli Hamidiye Etfal Training and Research Hospital, Istanbul, Turkey

## Abstract

Isolated avulsion fractures of the lesser tuberosity of the humerus are rare and can be difficult to diagnose. This study presents a fully arthroscopic technique for anatomic reduction and screw fixation of these injuries. The described method allows precise visualization, fragment control, and secure fixation using standard arthroscopic portals and equipment. It also allows simultaneous assessment and treatment of concomitant intra-articular pathologies. This technique offers a minimally invasive alternative to traditional open surgery, providing advantages such as reduced postoperative pain, minimal scarring, and faster rehabilitation.

Isolated lesser tuberosity (LT) fractures are rare and account for approximately 2% of proximal humeral fractures.^1^, ^2^, ^3^ They are usually seen in young patients. The annual incidence is 0.46 per 100,000 persons, with a median age of 43 years.^4^ The upper two-thirds tendinous portion of the subscapularis tendon attaches directly to the LT. The muscular portion of the lower third attaches inferior to the LT and to the anterior metaphysis of the humerus.^5^

The classic mechanism of injury is abduction and external rotation trauma of the shoulder. The subscapularis muscle contracts suddenly and strongly in this position, causing avulsion of the LT.^6^, ^7^, ^8^ Clinically, anterior shoulder pain and positive bear-hug, lift-off, and belly-press test findings are observed. Small or minimally displaced fractures may not be visible on standard radiographs.^8^^,^^9^ These fractures should be considered especially in patients with anterior shoulder pain and subscapularis muscle weakness after a fall.^10^ Advanced imaging modalities such as axillary radiography, computed tomography (CT), magnetic resonance imaging (MRI), and ultrasound are important in the diagnosis and determination of the degree of displacement.^9^ In cases in which the diagnosis is missed, this can lead to nonunion and eventually chronic shoulder pain and muscle weakness.^10^

Undisplaced fractures can heal successfully with conservative treatment.^6^^,^^8^ However, greater than 5 mm of displacement or greater than 45° of angulation is considered a criterion for surgery.^11^ According to some sources, even minimal displacement is an indication for surgery.^4^^,^^12^ Whereas the traditional approach is open reduction and fixation with screws, there is not enough information in the literature about arthroscopic techniques.^4^^,^^6^^,^^8^^,^^11^ This technical note describes a minimally invasive arthroscopic approach for fixation of LT fractures using cannulated screws (Video 1).

## Surgical Technique

### Preoperative Evaluation

**Fig 1:**
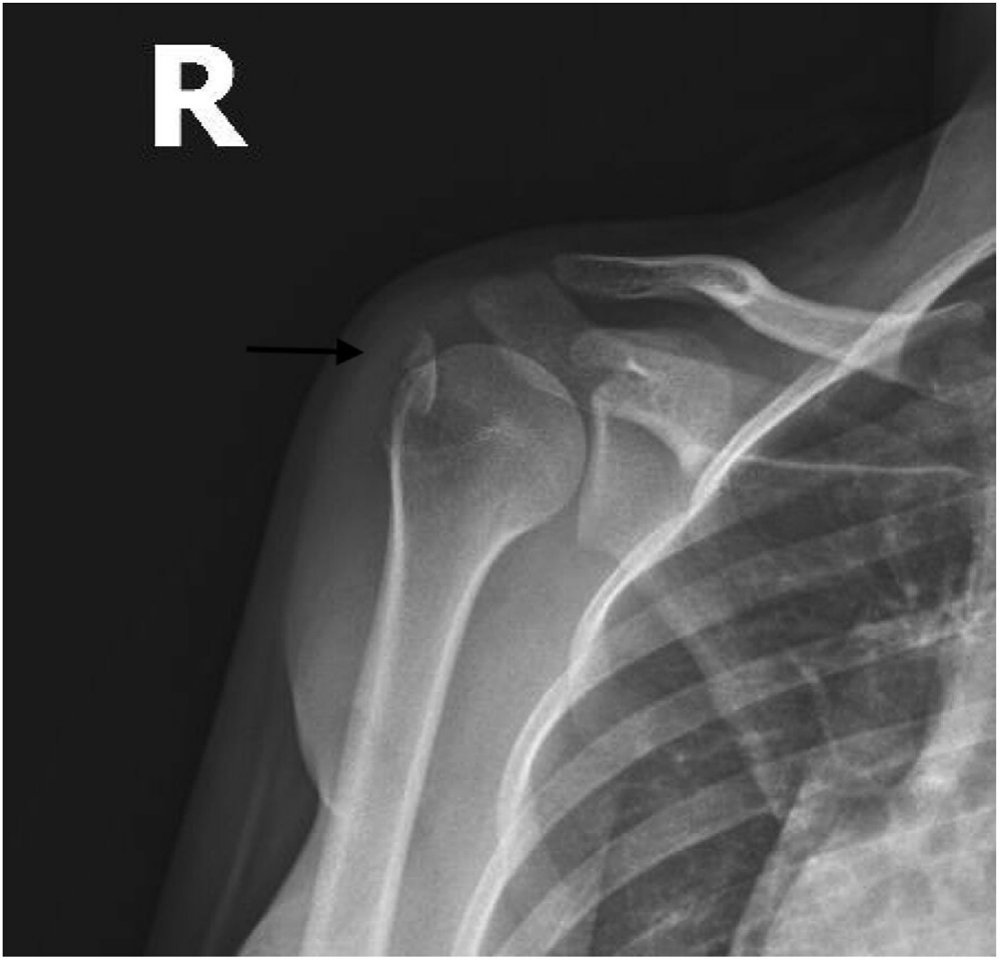
Anteroposterior radiograph showing a lesser tuberosity avulsion fracture (arrow). (R, right shoulder).

**Fig 2:**
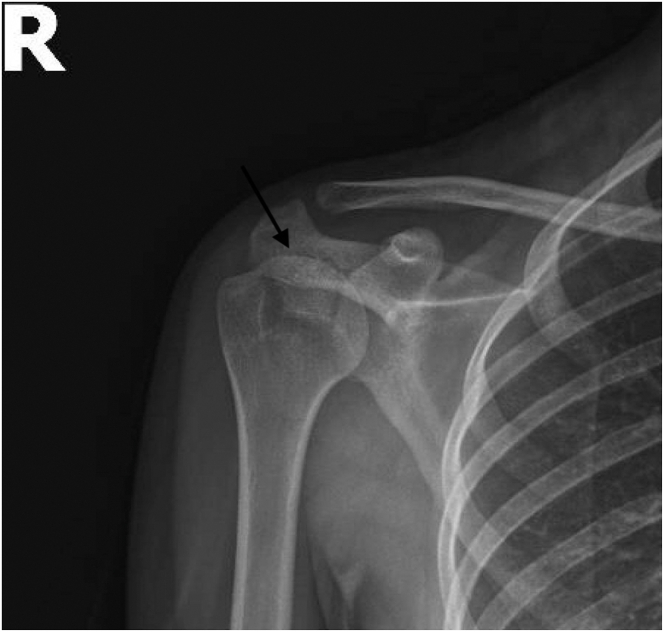
Anteroposterior radiograph showing a lesser tuberosity avulsion fracture (arrow). (R, right shoulder).

**Fig 3:**
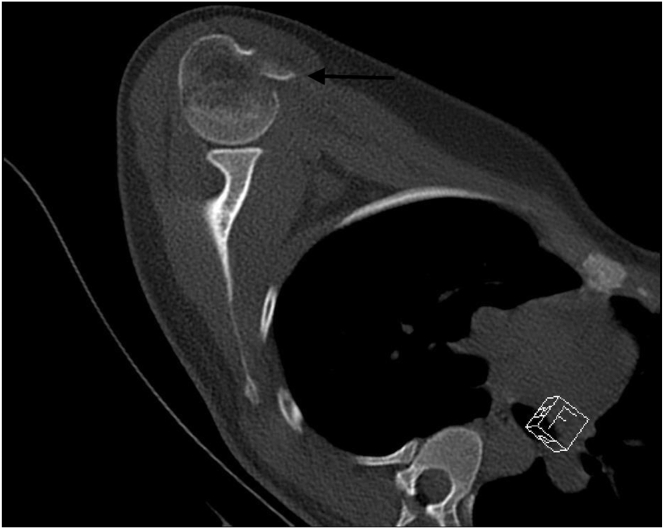
Axial computed tomography image showing a clearly displaced avulsion fragment of the lesser tuberosity (arrow). (R, right shoulder).

**Fig 4:**
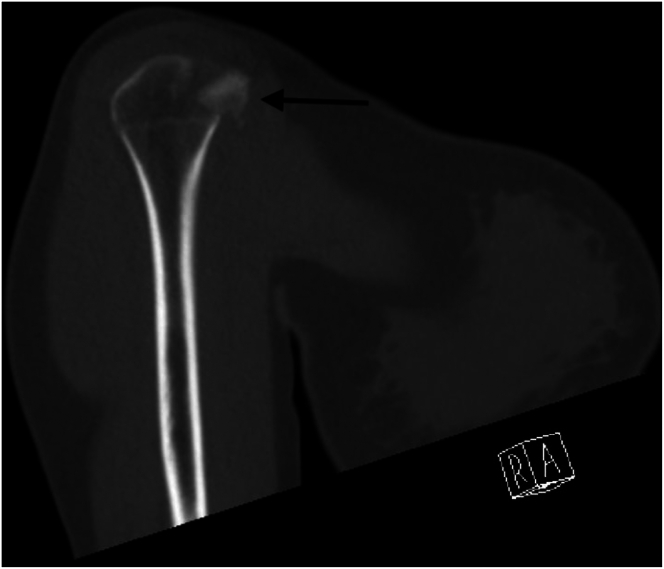
Coronal computed tomography image revealing fracture morphology and displacement of the lesser tuberosity fragment (arrow). (R, right shoulder).

Standard anteroposterior, scapular Y, lateral, and axillary radiographs are taken preoperatively (Figs 1 and 2). However, it is often difficult to understand the type of fracture from radiographs alone and an MRI or CT scan is usually required. MRI and CT are often necessary to understand the fracture structure. MRI provides information about the condition of the rotator cuff and soft tissues, whereas CT is useful in assessing the bone stock and fracture fragment structure (Figs 3 and 4). Appropriate surgical indications should be communicated to the patient after the evaluation of current images, considering that the planned surgical procedure may not be performed, with the possibility of a transition to open surgery; the necessary equipment for subscapularis repair should be available before surgery.

### Operating Room Setup

The patient’s identity, side, and imaging are confirmed. An interscalene nerve block is administered, followed by general anesthesia. A full examination of the shoulder is performed with the patient under anesthesia. The patient is placed in the beach-chair position, and the arm is prepared with sterile drapes.

### Arthroscopic Approach

A standard posterior portal is created with a 30° arthroscope (Smith & Nephew, Andover, MA) 2 cm medial and 2 cm distal to the posterolateral border of the acromion. Then, the anterior portal is opened. The joint structures are evaluated with diagnostic arthroscopy. The subacromial space is accessed through the posterior portal. The anterosuperior working portal is opened with direct vision. After bursectomy, a posterolateral portal is created for visualization and a midlateral portal is created for reduction.^13^

The glenohumeral joint is diagnostically evaluated with a 30° arthroscope through the posterior portal. With the help of a spinal needle, an anterior portal is created via the appropriate angle. During the arthroscopic evaluation, the biceps tendon may be subluxated or dislocated; the integrity of the subscapularis tendon and the humeral insertion sites is carefully examined.

In cases in which the biceps tendon is subluxated, displacement of the tendon and inadequacies in its stabilization may lead to failure of the subscapularis repair in the future. Therefore, biceps tenotomy or tenodesis is recommended, taking into account patient-specific factors.^14^^,^^15^

After the intra-articular evaluation is completed, the arthroscope is directed from the posterior portal to the subacromial space. An anterosuperior working portal is created under direct vision. Then, the field of view is widened by bursectomy. A posterolateral portal is opened for visualization, and a midlateral portal is opened for reduction procedures.

If accompanying rotator cuff or labral pathology is present, this may lead to narrowing of the subcoracoid space owing to arthroscopic fluid extravasation and iatrogenic swelling. Therefore, surgical priority should be given to reduction and fixation of the LT.^14^

### Fracture Reduction and Fixation

The subacromial space is entered through the posterior portal, and bursectomy is performed for visualization. The posterolateral portal is used as the primary arthroscopic imaging portal. At this stage, the biceps tendon and sheath are evaluated. The adhesions surrounding the fracture fragment and the relation of the subscapularis tendon to the fragment are observed. Necessary loosening and debridement procedures are performed through the anterior and anterolateral portals. The anterolateral and lateral portals are used for fracture reduction.

In chronic cases, if a long time has elapsed since the fracture occurred, the bone bed should be prepared with the help of a shaver and burr and the chondral tissues surrounding the fragment should be adequately debrided. A well-bleeding bone bed in this area promotes bone-on-bone healing and increases the success of reduction.

**Fig 5:**
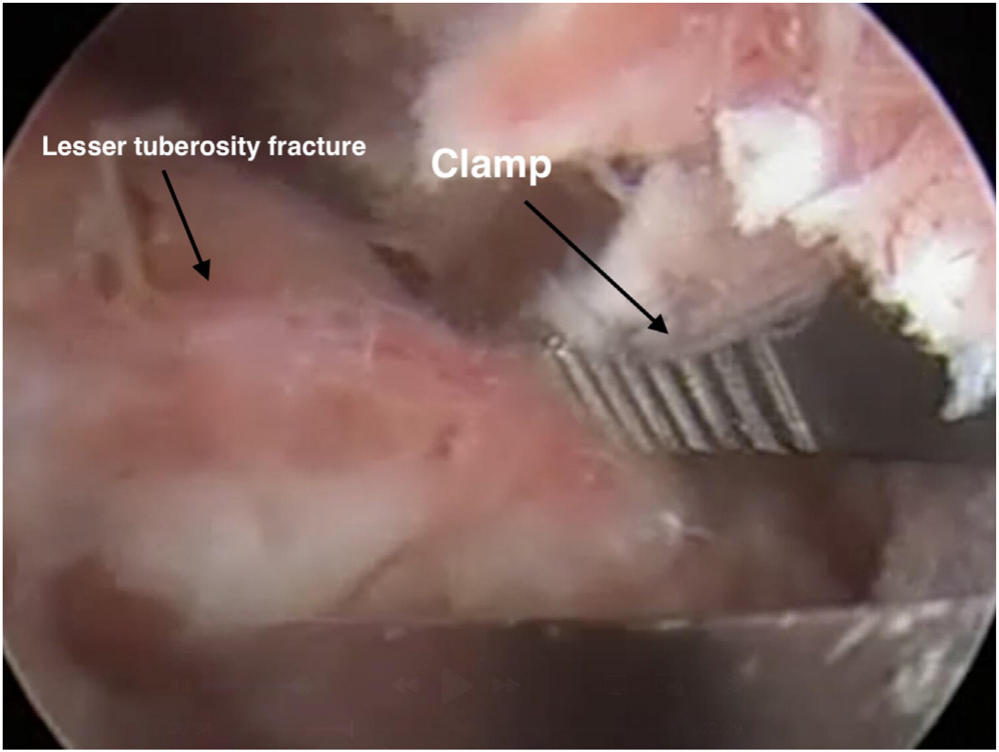
Arthroscopic view showing the lesser tuberosity fracture fragment (arrow) being grasped and reduced with a clamp (arrow). (Beach-chair position; posterior portal) (R, right shoulder).

If the LT fracture fragment is found to be rotationally displaced or in a nonanatomic position during arthroscopic evaluation, the fragment is gently grasped with the help of a clamp through the lateral portal, rotated to the anatomic position, and placed in the correct position (Fig 5). For stabilization, temporary fixation is provided through the anterolateral portal by passing through the marginal areas of the fragment with a number of K-wires appropriate to the size of the fractured fragment. The adequacy of the reduction is evaluated arthroscopically; fluoroscopic confirmation can be performed if necessary.

**Fig 6:**
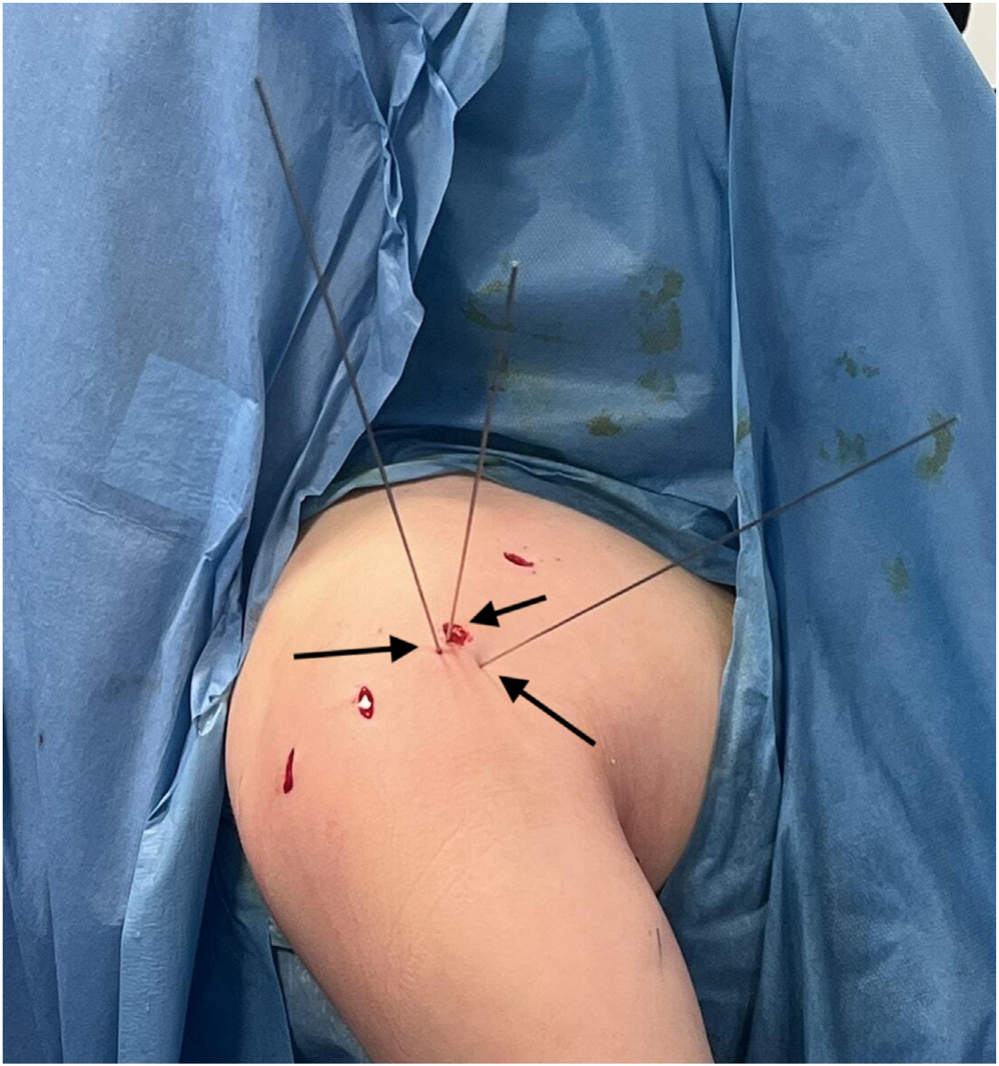
Intraoperative view showing temporary fracture stabilization using multiple percutaneous K-wires (arrows) during arthroscopic fixation of the lesser tuberosity. (Beach-chair position) (R, right shoulder).

**Fig 7:**
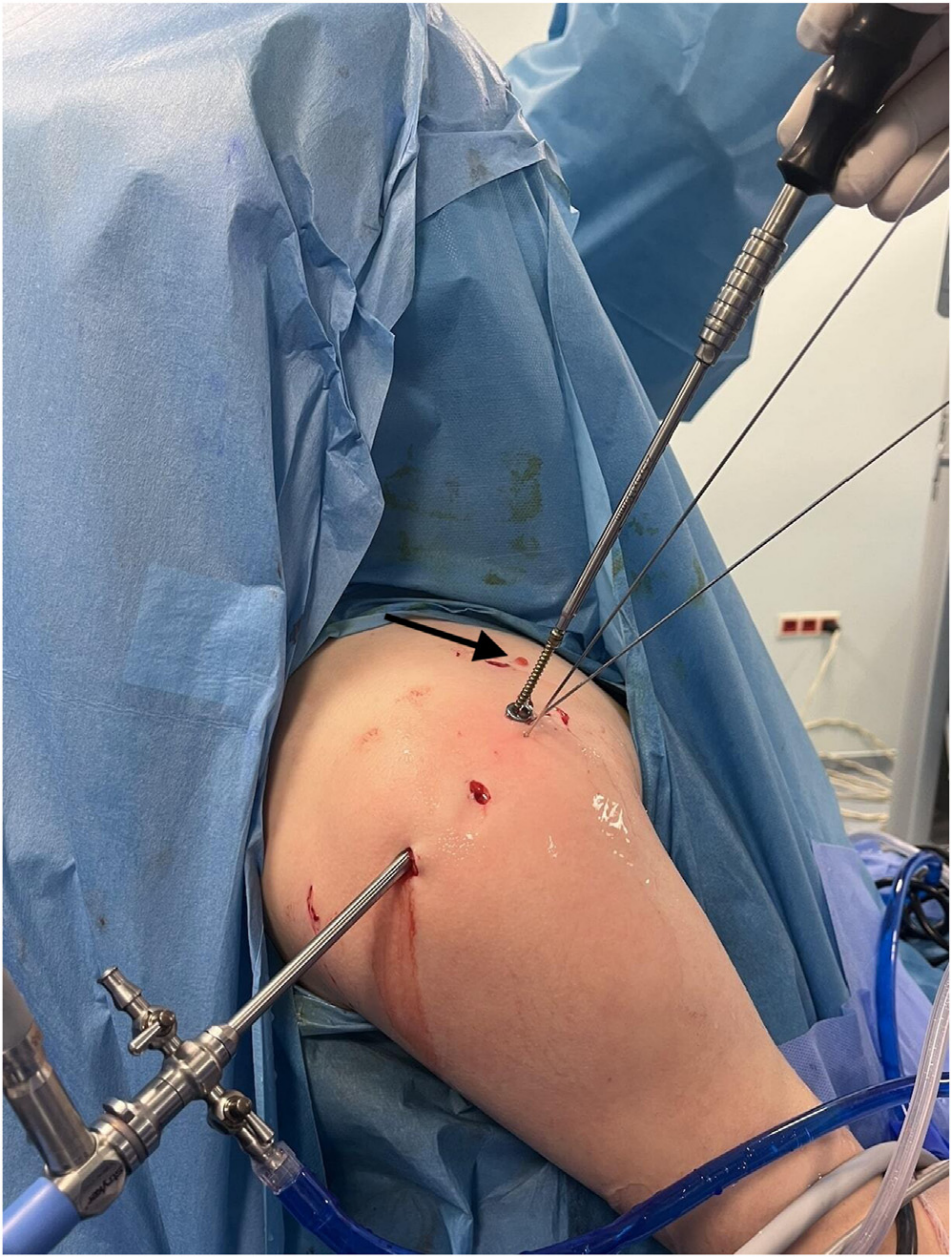
Intraoperative image showing fixation with a cannulated screw over a guidewire (arrow) under arthroscopic and fluoroscopic guidance. (Beach-chair position) (R, right shoulder).

**Fig 8:**
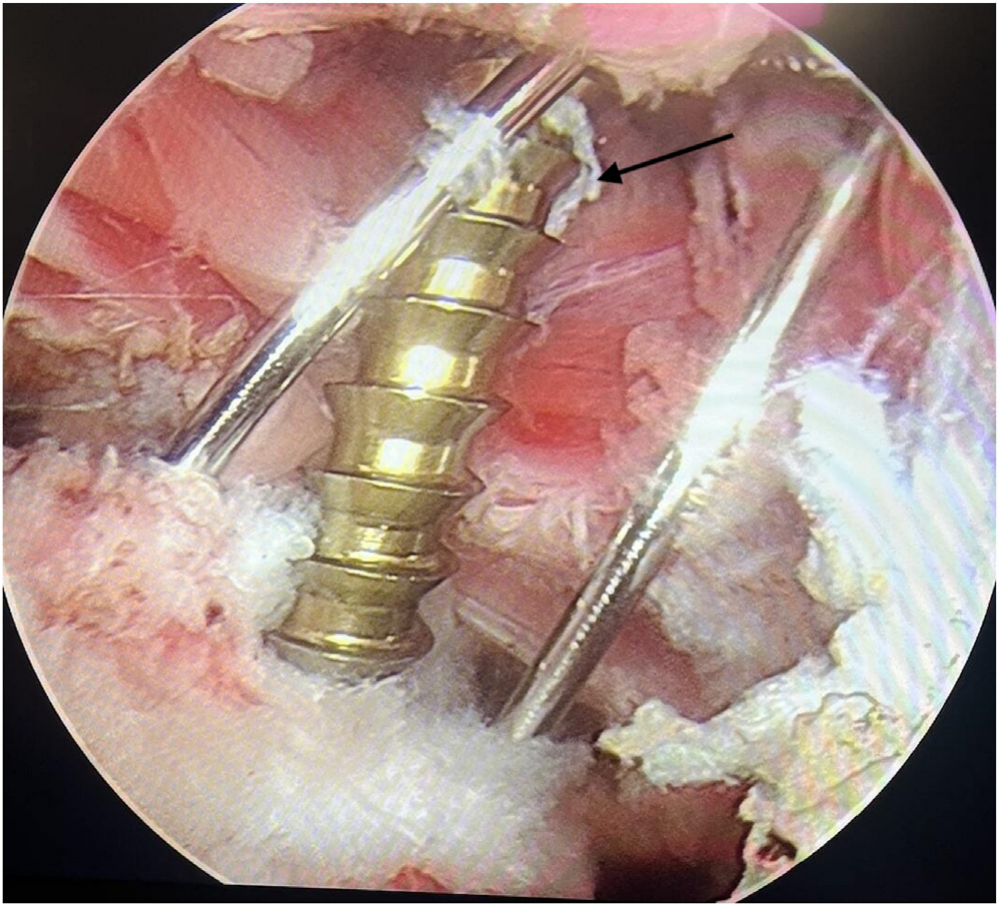
Arthroscopic view showing final fixation of the lesser tuberosity fragment with a cannulated screw (arrow) and supporting K-wires in place. (Beach-chair position; posterior portal) (R, right shoulder).

K-wires placed at the fracture site may cause pressure of the deltoid muscle and surrounding soft tissues on the fragment. In this case, the soft tissues are removed from the fracture with the help of a clamp through the lateral portal. Then, a cannulated K-wire is passed through the center of the fragment and fixation is provided with 1 or 2 cannulated screws (TST Medical Devices, Istanbul, Turkey) depending on the size of the fracture (Fig 6, Fig 7, Fig 8). When deemed necessary, washers can be used together with screws.

**Fig 9:**
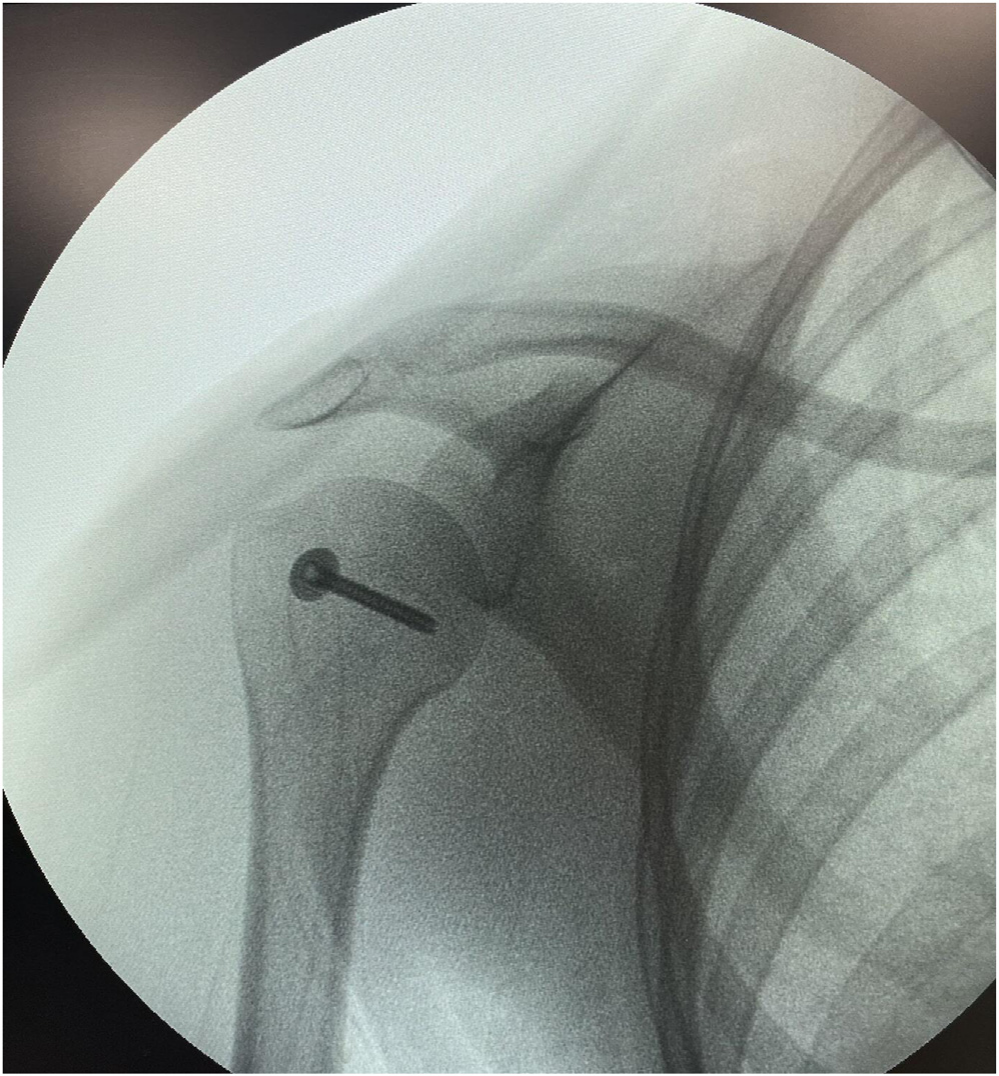
Intraoperative fluoroscopic image confirming anatomic reduction and proper placement of the cannulated screw in the lesser tuberosity. (R, right shoulder).

After the fixation is completed, the anatomic position and stability of the fractured fragment are confirmed arthroscopically. The K-wires are withdrawn, and the stability of the fragment is checked with the help of a probe. Finally, screw placement and the condition of the fracture line are evaluated with fluoroscopy (Fig 9). After the surgical procedure, the patient is examined and the presence of additional pathologies is investigated.

### Postoperative Care and Rehabilitation

An arm sling is used for 3 weeks. Elbow and wrist movements can be started on the first day after surgery. On day 3, passive shoulder movements are started, limited to 90° of flexion and 60° of abduction and internal rotation. Active range of motion is permitted in weeks 4 through 6. Strengthening starts in the third month.

## Discussion

Avulsion fractures of the LT are rare injuries that are often difficult to diagnose.^4^^,^^16^ They usually occur as a result of high-energy trauma such as falls from stairs, cycling accidents, or falls during horse riding. MRI or CT may be required in patients with such injuries because they may not be detected on standard radiographs. Even if the fracture shows minimal displacement, displacement of the fragment is often an indication for surgery.^17^

Although there are studies showing successful results with conservative treatments, these approaches carry the risk of complications such as nonunion, loss of rotation, pain, and decreased muscle strength.^8^^,^^11^ Traditionally, treatment via open reduction and internal fixation was preferred for LT avulsion fractures, but nowadays, with the development of arthroscopic techniques, interest in minimally invasive methods is increasing.

Compared with open surgery, arthroscopic surgery has the advantages of being less invasive, leaving minimal skin scarring, causing theoretically less postoperative pain, and allowing a shorter rehabilitation period. It is also an important advantage that accompanying structural lesions such as rotator cuff tears or labral pathologies can be recognized and treated arthroscopically in the same session. In a study by Lin et al.,^18^ it was reported that 40% of cases of avulsion fracture of the LT of the humerus had concomitant pathologies. For this reason, arthroscopic repairs are increasingly being accepted owing to reduced morbidity and similar clinical outcomes. A meta-analysis by Vavken et al.^19^ showed that open and arthroscopic surgical procedures yield similar functional results.

In the traditional surgical approach, open reduction and fixation with screws are recommended for large fragments,^6^^,^^8^^,^^11^^,^^16^ whereas for smaller bone fragments, fixation using an anchor with arthroscopic methods is preferred.^16^^,^^20^, ^21^, ^22^ Alternatively, LT excision can be performed in some cases.^23^ When a subscapularis tendon tear is present, arthroscopic repair of the tendon after excision is also a common method.^5^^,^^14^^,^^15^^,^^24^ In this study, the feasibility of completely arthroscopic application of the classic open screw fixation technique is shown.

Anatomic structures to be considered during the aforementioned surgical procedures include the neurovascular bundle passing just medial to the coracoid and the axillary nerve traveling in the neighborhood of the LT and subscapularis tendon. Because these structures are vulnerable to damage during surgery, the procedure should be performed in a careful and controlled manner. The presented arthroscopic technique offers a safe, predictable, and effective approach with standard arthroscopic equipment and surgical skills, providing rapid healing, early rehabilitation, and full functional return.

In conclusion, a high clinical index of suspicion is important in cases of avulsion fractures of the LT after high-energy trauma because of their rarity and frequently overlooked presentation. Surgical intervention is usually necessary, especially for displaced fractures, and arthroscopic fixation offers effective results as a minimally invasive alternative.

## Disclosures

Both authors (E.İ., Y.S.) declare that they have no known competing financial interests or personal relationships that could have appeared to influence the work reported in this paper.

## Supplementary Data

Video 1 Arthroscopic screw fixation technique for an isolated lesser tuberosity avulsion fracture. The procedure is performed on the right shoulder with the patient in the beach-chair position. Diagnostic arthroscopy is initiated through a posterior viewing portal, and a standard anterior working portal is established. A comprehensive evaluation of the glenohumeral joint is performed. The avulsion fracture site is then identified and prepared. Anatomic reduction of the fragment is achieved using an arthroscopic grasper. Final fixation is performed using cannulated screws under combined arthroscopic visualization (posterior portal) and fluoroscopic guidance, ensuring accurate positioning and stable fixation of the fragment.
